# On the Absorption of Alimentary Substances, and the Functions of the Lacteals

**Published:** 1851-10

**Authors:** 


*On the Absorption of Alimentary Substances, and the Functions of the Lacteals.* By M. C. Bernard. (*Gazette Medicale.*) M. Bernard read a memoir on this subject, in which he proposed to determine, by direct experiment the nature of the nutritive principles which are absorbed and conveyed by the chyliferous vessels, in order to ascertain if there really exist any alimentary substances which absolutely escape venous absorption, and consequently avoid passing through the liver before arriving at the lungs.

Alimentary substances submitted to digestion are, in the intestinal canal finally reduced to three principal substances — the saccharine, the albuminous, and the fatty emulsive; on these M. Bernard has instituted experiments:

1. *With Regard to the Absorption of Sugar by the Lacteals.*—On injecting large quantities of sugar into the stomachs of different mammiferæ, it has been found in the blood of the portal vein, while it was absent from the chyle of the thoracic duct at the same time and under the same circumstances; whence it is concluded that sugar, before arriving at the lungs, traverses the liver, where it undergoes a peculiar physical modification. If a solution of grape sugar be injected into the superficial veins of a dog, it speedily passes off by the urine; on the contrary, if the solution of sugar be injected into the radicles of the portal vein, the sugar is no longer eliminated by the kidneys, but passes into the circulation, and is assimilated in the same manner as if taken into the digestive canal. Thus it is shown that the absorption of sugar by the portal system is a condition essential to its assimilation, since, if confined to the lacteals, the saccharine principle is abstracted from the influence of the liver, and is diverted directly into the general venous circulation, as takes place when it is injected by the jugular vein.

2. *As to the Absorption of Albumen by the Lacteals.* — Albumen injected into the general venous circulation soon appeared in the urine. If injected into the portal vein, it does not then appear in the urine, but is assimilated in the same manner as obtains with sugar.

3. *Absorption.* — M. Bernard's previous researches have shown that fatty matters are not capable of admission into the lacteals until an emulsion has been formed by an action of the pancreatic juice. Immediately that this emulsion has penetrated the lacteals, their aspect undergoes an entire change; instead of remaining transparent, like other lymphatics of other parts of the body, they assume a milk-white appearance, and, owing to the transparency of the coats of these vessels, the course of the fatty matter may be followed from the intestine to the subclavian vein, where it is diverted into the thoracic duct. It is not necessary that fatty matters should traverse the liver in order to their assimilation. M. Bernard has injected fatty emulsions into the jugular vein, but has not found that substance in the urine.

Thus the products of digestion may be distinguished, with reference to absorption, into two groups — *e. g.*, First, fatty and albuminous matter absorbed by the lacteals, passing into the general circulation without having traversed the liver. The last proposition cannot be taken in so absolute a sense as the former, since experiment and microscopical examination demonstrate that fatty matters are absorbed both by the portal system and by the lacteals. — *Charleston Medical Journal.*

